# Rice Husk as a Sustainable Amendment for Heavy Metal Immobilization in Contaminated Soils: A Pathway to Environmental Remediation

**DOI:** 10.3390/toxics12110790

**Published:** 2024-10-29

**Authors:** Riccardo Cecire, Aleandro Diana, Agnese Giacomino, Ornella Abollino, Paolo Inaudi, Laura Favilli, Stefano Bertinetti, Simone Cavalera, Luisella Celi, Mery Malandrino

**Affiliations:** 1 Department of Chemistry, University of Turin, Via Giuria 7, 10125 Turin, Italy; riccardo.cecire@unito.it (R.C.); stefano.bertinetti@unito.it (S.B.); simone.cavalera@unito.it (S.C.); 2 Department of Agricultural, Forest and Food Sciences, University of Turin, Largo Paolo Braccini 2, 10095 Grugliasco, Italy; luisella.celi@unito.it; 3 Department of Drug Science and Technology, University of Turin, Via Giuria 9, 10125 Turin, Italy; agnese.giacomino@unito.it (A.G.); ornella.abollino@unito.it (O.A.); paolo.inaudi@unito.it (P.I.); laura.favilli@unito.it (L.F.)

**Keywords:** soil remediation, soil amendment, broadleaf plants, potentially toxic elements, Tessier protocol, pot experiments

## Abstract

Rice husk is a waste byproduct of rice production. This material has a moderate cost and is readily available, representing 20–22% of the biomass produced by rice cultivation. This study focused on the properties of rice husk in the remediation of soils contaminated by heavy metals. The effect of particle size, pH, and the presence of organic ligands on sorption efficiency was evaluated for Cd, Cu, and Mn. The continuous flow method was used to select suitable operative conditions and maximize the retention of heavy metals. Subsequently, pot experiments were carried out by growing two broadleaf plants, *Lactuca sativa* and *Spinacia oleracea*, in aliquots of soil collected in a Piedmont (Northwest Italy) site heavily contaminated by Cu, Cr, and Ni. Rice husk was added to the contaminated soil to evaluate its effectiveness in immobilizing heavy metals. The availability of Cr, Mn, Ni, Cu, Zn, Cd, and Pb in soil was studied using Tessier’s sequential extraction protocol. The content of the elements was also analyzed in plants and the uptake of heavy metals was evaluated in relation to the addition of rice husk. The growth of both plants was more efficient in the presence of rice husk due to its ability to reduce the mobility of heavy metals in the soil. The simplicity, cost-effectiveness, and scalability of its employment make the use of rice husk suitable for practical applications in soil remediation.

## 1. Introduction

Food security is a critical issue for peri-urban areas, as they are vulnerable to contamination due to the coexistence of various anthropogenic activities, including industrial discharges, agricultural runoff, and atmospheric deposition [1,2,3]. The presence of heavy metals in peri-urban soils is of great concern because of their adverse effects on the environment, human health, and agriculture [4,5].

Plants can be overly sensitive to heavy metals, which can trigger various physiological responses even at low stress levels. While some heavy metals are essential for plant growth, high concentrations can cause toxicity, compromising physiological processes and posing a risk to food safety [6]. The effects of heavy metal contamination on plants are complex and depend on the specific metal; for instance, Cd and Pb affect respiration and photosynthesis, while Zn and Cu disrupt cell function and membrane integrity [6]. The mobility and availability of heavy metals to plants are contingent upon soil characteristics, including mineral composition, organic matter content, pH, and redox potential [2].

Heavy metals, in contrast to organic pollutants, exhibit remarkable persistence in soils as they resist microbial or chemical degradation [3]. Increases in public awareness about the health consequences of contaminated soils have promoted the creation of innovative approaches to their remediation, especially considering the prohibitive costs associated with traditional methods [1].

Selecting the most suitable remediation technology hinges on several critical factors, including the type and extent of contamination, the economic feasibility, and the overall efficiency of the chosen method. In peri-urban areas, the complexity of contamination sources and land use patterns necessitates a careful evaluation of these factors to determine the optimal approach. Efficiency is a paramount consideration when choosing remediation technology, and it is essential to assess whether the method can effectively reduce the bioavailability of heavy metals in the soil, thus minimizing risks to human health and the environment. Economic feasibility is another critical factor, and the cost of implementing remediation technology, including material acquisition, pre-treatment cost, labor, and maintenance, must be balanced against the potential benefits and long-term impacts [7].

The remediation of contaminated soils involves a range of approaches that can be broadly categorized into physical, chemical, and biological methods [8,9]. Each category has its unique advantages and limitations, making the selection of the most appropriate technique contingent on the specific characteristics of the contamination and the environmental context. Chemical methods, which encompass various techniques for altering the chemical properties of the soil, have shown promising results in immobilizing contaminants and reducing their bioavailability [10,11]. In order to achieve this objective, various soil amendments, such as phosphate compounds, liming materials, organic matter residues, metal oxides, and biochar, are currently studied as options for soil remediation [12]. These amendments interact with heavy metals in the soil and transform them into less mobile and less bioavailable forms.

Rice husk is an environmentally friendly and readily available material, representing 20–22% of the biomass produced by rice cultivation. It is rich in silica (20%), which has a high affinity for heavy metals due to the existence of ionizable silanol groups. Furthermore, rice husk contains hemicellulose (12%) and proteins (3%) that, with carboxyl and amine groups, can give interactions with heavy metals. Rice husks also contain organic acids such as oxalic, citric, acetic, malic, and succinic, which can form stable complexes with heavy metals [13]. All these components in rice husk can facilitate the adsorption and complexation of heavy metals in soil, reducing their mobility and bioavailability. The low management costs and the fact that it is a waste product make it a cost-effective solution for soil remediation. Additionally, utilizing rice husk for soil remediation exemplifies the principles of waste upscaling and valorization, transforming an agricultural byproduct into a valuable resource. This approach not only mitigates waste disposal issues but also provides an innovative and sustainable method for addressing soil contamination [14].

Furthermore, rice husk-derived materials have garnered attention for their remarkable adsorption properties and their potential for immobilizing heavy metals in wastewaters and soil [1,15,16]. For instance, Zheng et al. (2012) explored the use of biochar derived from rice residue to assess its impact on heavy metal accumulation in rice seedlings [17]. It was found that while biochar effectively reduced the uptake of Cd, Zn, and Pb by rice plants, it increased the accumulation of As. This dual effect underscores the complexity of biochar application in agricultural settings. In addition, Derakhshan Nejad and Jung (2017) focused on rice husk biochar and its efficacy in immobilizing multiple heavy metals (Cu, Cd, Zn, Pb) in soil, highlighting rice husk biochar as a promising soil amendment for reducing heavy metals’ phytoavailability and leachability [18]. Furthermore, Bian et al. (2022) conducted pot experiments to evaluate the potential of rice husk-derived biochar to mitigate Cd uptake by cabbage leaves. Results indicated that biochar application significantly decreased Cd uptake, demonstrating its potential to enhance food safety and soil health [19].

Despite the substantial research focused on treated rice husk materials that show promising results in heavy metal remediation, there remains a notable gap in understanding the potential efficacy of untreated rice husk in this context. This underscores the necessity to explore the inherent properties of untreated rice husk without the additional costs associated with treatment processes, particularly in scaling up its application for soil remediation purposes.

In the present work, we have delved into the scientific principles behind rice husk’s effectiveness as a soil amendment, its practical application, and the feasibility of implementing this approach in peri-urban areas.

This paper will discuss the characterization and feasibility of rice husk as an adsorbent of heavy metals (namely Cd, Cu, and Mn) as a function of the effect of particle size, pH, and the presence of organic ligands. Subsequently, the changes in metal availability in the soil after application of rice husk were studied. Finally, its application as a soil amendment in a heavy metal-contaminated soil sample was evaluated as well as its effectiveness in reducing the uptake of the pollutants by two plants, *Lactuca sativa* and *Spinacia oleracea*.

In the present work, the aim was to evaluate the potential of untreated rice husk as an amendment to reduce the bioavailability of heavy metals in contaminated soils. This was achieved by (1) characterizing the sorption behavior of rice husk towards heavy metals (Cd, Cu, Mn) based on particle size, pH, and the presence of organic ligands; (2) studying the changes in metal availability in soil after rice husk application; and (3) evaluating its effect on reducing heavy metal uptake by *Lactuca sativa* and *Spinacia oleracea.* The hypothesis is that untreated rice husk can effectively decrease the availability of heavy metals in soil because of its abundant silica and organic compounds content, offering an affordable and eco-friendly method for remediation.

## 2. Materials and Methods

### 2.1. Apparatus and Reagents

For acid digestion, an MLS-1200 MEGA microwave (Milestone, Sorisole, Italy) oven was used, equipped with a sample carousel with Teflon vessels fitted with a safety valve closure.

The concentration of the analytes in the tested solutions was determined by inductively coupled plasma atomic emission spectroscopy (ICP-AES) using a VARIAN LIBERTY 100 spectrometer (Varian Australia, Mullgrave, Australia). The wavelengths of the analytes chosen and the limits of detection (LOD) are present in Table S1.

Due to their lower concentration, Cd and Pb were also determined by graphite furnace atomic absorption spectrometry (GF-AAS) with a Perkin Elmer 5100 instrument (Perkin Elmer, Norwalk, CT, USA).

Determinations of the metals were performed using external calibration. The calibrations were always performed with standard solutions prepared in aliquots of sample blanks (matrix matching method). Metal ion standards were prepared from commercial 1000 mg/L stock solutions (MERCK Titrisol, Darmstadt, Germany; vials containing 1.000 ± 0.002 g metal in salt form). The reagents used are all analytical purity grade. All solutions were prepared using HPW (High Purity Water). Water was purified in a Milli-Q system, resulting in water with a resistivity of 18 MΩ⋅cm.

A pH meter, model EA 920, from the company ORION (Orion Research INC, Ayer, MA, USA) was used to measure the pH of solutions, equipped with a glass/calomel combination electrode. The instrument was calibrated daily using two standard solutions, one at pH 4 (citrate-HCl) and one at pH 7 (NaH_2_PO_4_-Na_2_HPO_4_). The determination is subject to an uncertainty of 0.02 pH units [20].

**Figure 1:**
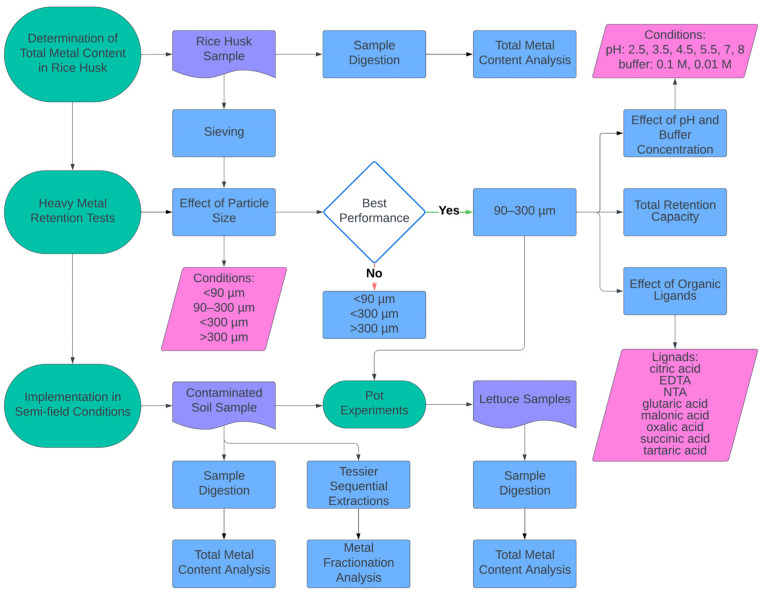
Block diagram of experiments performed.

In Figure 1, a block diagram is illustrated that shows the experimental workflow of this study.

### 2.2. Determination of Total Metal Content in Rice Husk

Rice husk was obtained from a farm in the province of Pavia (Lombardy, Northwest Italy). The inorganic content of the rice husk was characterized to assess the possible release of heavy metals during retention tests and to evaluate the possible input of contaminants into the soil. Three sample aliquots of 0.1 g of untreated rice husk were digested with 5 mL of aqua regia and 2 mL of hydrofluoric acid (HF). The following heating program was applied in the MW oven: four heating steps of 5 min each (250, 400, 600, 250 W, respectively). Subsequently, 0.7 g of boric acid (H_3_BO_3_) were added, and the samples were heated again for 5 min at 250 W. The resulting solutions were filtered with paper filters and diluted to 100 mL with HPW. The solutions were analyzed by ICP-AES.

### 2.3. Heavy Metal Retention Tests

**Figure 2:**
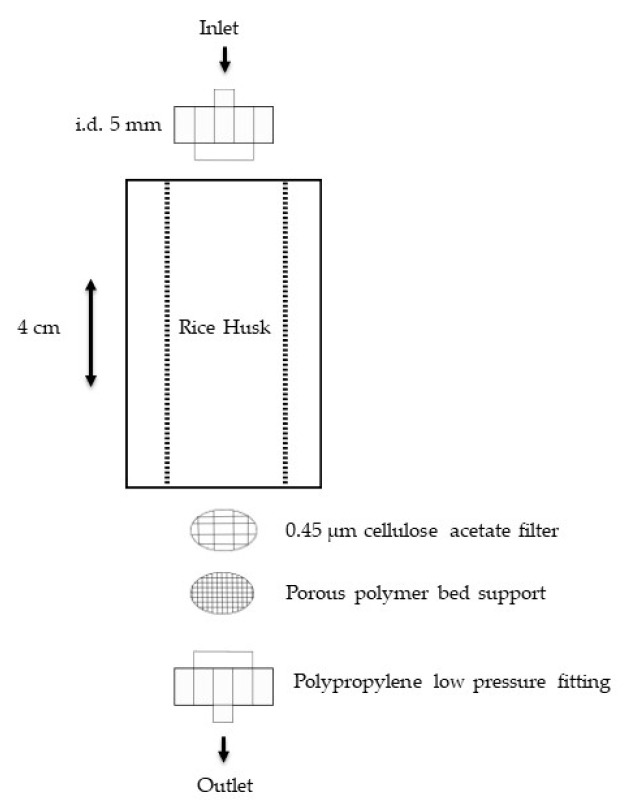
A schematic representation of the continuous flow system apparatus used for the experiments.

A continuous flow system was employed (Figure 2) in preference of the batch technique to understand the effect of various considered parameters (particle size, pH, organic ligands) on the retention of metals by the rice husk, because this system can be easily scaled up from a laboratory system to a real application [21,22]. This system was preferred to better simulate a dynamic equilibrium condition of a soil subject to frequent environmental stresses than a static equilibrium situation.

Metal adsorption tests were conducted in polypropylene columns (Bio-Rad, Hercules, CA, USA; length 4 cm, internal diameter 5 mm) packed with 0.25 g of rice husk. At the bottom of the column, a cellulose acetate filter (Millipore Merck, Darmstadt, Germany) was placed on the porous polymer support to prevent clogging of the porous septum and the loss of the smallest rice husk particles into the eluates. The slurry packing method was used to obtain a homogeneous bed of particles in the column. The method consists of placing rice husk and 5 mL of HPW in a beaker to create a suspension.

Standard solutions of the metals under investigation were injected through the columns with the help of a Gilson Miniplus 2 (Gilson Middleton, CT, USA) multichannel peristaltic pump at a constant flow rate (0.5 mL/min). The pump was connected to the columns with polypropylene connectors and PVC tubing (i.e., 1.29 mm). Before each experiment, rice husk was conditioned to the working pH by flowing the proper buffer solution. Solutions of the metal ions at a concentration of 1 × 10^−3^ M were prepared and diluted with buffer solutions at the desired pH. The following buffer solutions were utilized: trichloroacetic acid (TCA) buffer, utilized for experiments with pH values less than 3; acetic acid/sodium acetate buffer (HAc/NaAc), utilized for experiments conducted at pH 3.3–5.5; HEPES buffer, utilized for pH 7 and 8 experiments.

Column retention experiments were conducted on untreated rice husk monitoring three metal ions: Cu, Cd, and Mn. Three replicates of each experiment were performed. For each experiment, a procedural blank was also performed to account for contamination or release of the metals from the rice husk. Finally, 25 mL of the eluate were collected and analyzed by ICP-AES. The amount of metal retained on the rice husk was calculated by the difference between the metal content in the input and output solution, corrected for the value of the blank, and expressed as a percentage.

#### 2.3.1. Effect of Particle Size

Rice husk contains particles of assorted sizes, ranging from small to coarse. Since it must be packed into columns, it is essential to have a sort of size uniformity, avoiding preferential paths. Therefore, the choice of particle size was crucial to improve the interaction with metal ions. Tests were conducted at pH 5.5 with a concentration of 0.1 M acetate buffer, 1 × 10^−4^ M metal ions (Cd, Cu, and Mn), and four particle size fractions of rice husk obtained after sieving: <90 µm; between 90 and 300 µm; <300 µm; >300 µm. The behavior of the metals was studied both individually and in mixtures.

#### 2.3.2. Effect of pH and Buffer Concentration

Metal adsorption depends on numerous factors including pH and ionic strength. Industrial and processing waters and wastewaters, as well as contaminated soils, show often a broad range of pH values and behave differently toward divalent cations in competition with one another [23]. For these reasons, the effect of pH (2.5–8) and the buffer concentration (1 × 10^−1^ M and 1 × 10^−2^ M) on the retention of Cd, Cu, and Mn by rice husk was investigated. Experiments were conducted with metals concentration of 1 × 10^−4^ M in mixtures.

#### 2.3.3. Effect of Ligands

The retention of metals by rice husk can be affected by the presence of ligands, contingent upon the properties of the ligand, rice husk, and metal under consideration. In this study, we studied the effect of following organic ligands: citric acid, ethylenediaminetetraacetic acid (EDTA), nitrilotriacetic acid (NTA), glutaric acid, malonic acid, oxalic acid, succinic acid, and tartaric acid. These are some of the most used ligands for metal complexation and their functional groups were used to simulate the behavior of more complex organic molecules potentially present in soils. The metal ion concentration was 1 × 10^−4^ M, and the ligand concentration was 3 × 10^−4^ M. The effect of the presence of ligands on the retention of the three metals was conducted at pH 3.5 with a buffer concentration of 1 × 10^−2^ M. It was decided to simulate an extreme environmental condition with a low pH to better simulate the acidic effluents characterizing pollution of our case study.

To analyze the data obtained in the experiment on the presence of ligands, One-Way ANOVA by the XLSTAT software (version 2022.2.1) was used to check if there was a statistical difference between the mean concentrations in the effluent solutions of the nine groups for each metal investigated. The assumption of normality and homogeneity of variances was assessed prior to conducting ANOVA to ensure the appropriateness of the test [24]. Following the ANOVA, a Dunnett post hoc test was applied to compare each treatment group against a single control group, determining whether significant differences existed between the control and the treated groups. This approach helps the control for Type I errors associated with multiple comparisons, ensuring robust statistical conclusions [25].

PyES (version 1.1.3), an open-access software that allows for the calculation of the distribution of species in solution under the experimental conditions, was used [26].

#### 2.3.4. Total Retention Capacity

The total retention capacity of rice husk towards Cd, Cu, and Mn was studied in conditions of nearly quantitative uptake for all the metals to ensure that the amount of metal retained by the rice husk depends mainly on thermodynamic reasons. The saturation point was determined when the concentration of the influent solution (C_0_) equaled that of the effluent solution (C), indicating full occupation of the rice husk’s surface adsorption sites. The breakthrough curve was constructed by plotting the ratio of C/C_0_ as a function of the effluent volume [27]. The set-up is the same as described in the previous paragraph except for the type of columns used: ECONO-Column Chromatography Bio-Rad, diameter 1.5 cm, volume 18 ml (Bio-Rad Laboratories, Hercules, CA, USA). Solutions of the metal ions at a concentration of 1 × 10^−3^ M were prepared and diluted with acetate buffer in concentration of 1 × 10^−2^ M at pH 5.5.

### 2.4. Implementation in Semi-Field Conditions

We tested rice husk as a soil amendment for the remediation of contaminated soil. Contaminated soil samples were derived from the town of Borgomanero, in the province of Novara (Piedmont, Northwest, Italy). A map of the location is presented in Figure S1 in the Supplementary Material. The area was previously used as a permanent meadow and woodland in the past and became contaminated because of the repeated floods of a small stream collecting the wastewater of local industries, some of which operate in the electroplating processes. Its floods caused an accumulation of inorganic contaminants in the soil. The core of the contaminated zone was about 3000 m^2^ wide.

The soil sampling was originally conducted in 2012, following the work by Malandrino et al. (2011) [28]. Several sub-samples were collected across the contaminated site to ensure comprehensive coverage. These samples were properly stocked and preserved until their use in this experiment. For the current study, a composite soil sample was created by mixing the sub-samples collected during the 2012 sampling, ensuring homogeneity and representativeness of the contaminated site. This approach allowed for a consistent and well-characterized sample throughout the experiment, enhancing the reliability of the findings.

For the characteristics of the contaminated soil, see Table S2 and Malandrino et al. (2011) [28]. The contaminated soil texture is classified as loamy sand, and the soil pH is strongly acid (5.1–5.5) according to the USDA (2024) classification [29].

A control soil was also used. As shown in Table S2, the control soil was chosen based on its high organic matter content, substantial cation exchange capacity, and balanced texture to provide an ideal environment for the growth and development of lettuce and spinach, making it a suitable control for this experiment.

#### 2.4.1. Determination of Total Metal Content in Soil

Soil samples were collected with plastic tools and transferred into polyethylene bags. Subsequently, each sample was air-dried, sieved through a 2 mm sieve, ground in a centrifugal ball mill, and stored in plastic bags prior to laboratory analysis. Three soil sample aliquots of 0.5 g were treated with a mixture of 10 mL of aqua regia and 4 mL of HF in PTFE vessels and mineralized in a microwave oven. Four heating steps of 5 min each (250, 400, 600, and 250 W respectively) were used. Then, 1.4 g of H_3_BO_3_ was added, and the vessels were further heated for 5 min at 250 W and again cooled by ventilation for 25 min. The resulting solutions were filtered with paper filters and diluted to 100 mL with HPW. The solutions were analyzed by ICP-AES and GF-AAS.

#### 2.4.2. Tessier Fractionation

We evaluated how the addition of rice husk affected the mobility and reactivity of metals in soil using Tessier fractionation. This extraction procedure, adapted from Tessier et al. (1979), categorizes metals into five distinct chemical fractions: extractable and exchangeable, carbonate-bound, Fe and Mn oxide-bound, organic matter- and sulfide-bound, and residual [30]. The residual fraction is excluded as it is mainly integrated within the crystal structures of rocks and minerals, releasing only over the long term [31,32]. After each extraction, the suspension was centrifuged for 20 min at 4000 rpm. The supernatant was separated, while the solid residue was washed with 10 mL HPW and centrifuged again for 5 min. The washed water was added to the supernatant, while the solid residue was ready for the next extraction. The extracts were diluted to 25 mL (first fraction), 50 mL (second fraction), and 100 mL (third, fourth, and fifth fractions), stabilized by the addition of 25, 50, and 100 µL of concentrated HNO_3_, respectively, and analyzed. The concentrations of Cr, Cu, Mn, Ni, and Zn in the five fractions were determined by ICP-AES, whereas Cd and Pb were determined by GF-AAS.

#### 2.4.3. Effect on Lettuce and Spinach Heavy Metal Uptake

To evaluate the effectiveness of the rice husk in reducing the phytoavailability of the metal pollutants in a real scenario, we evaluated the effect of the addition of this material by measuring metal uptake by lettuce (*Lactuca sativa*) and spinach (*Spinacia oleracea*), used as test crops [33,34].

Pot experiments were made by putting untreated contaminated soil (5 kg) into polyethylene pots and adding rice husk (500 g). At the same time, an aliquot of the contaminated soil and one of unpolluted soil were left unamended and used as references. Each treatment was performed in triplicate. The experiment was conducted with two plant species, *Lactuca sativa* and *Spinacia oleracea*. The pots were laid out at room temperature (25 °C), and they were watered three times a week with 500 mL of tap water. Plants grown in the pots were harvested after 2 months.

All harvested plants were stripped of their roots, washed with HPW, oven-dried at 60 °C for 16 h, and then ground in an agate mortar. Then, 0.2 g of ground plant material was digested with 10 mL of concentrated HNO_3_ in a MW oven, using four heating steps of 5 min each (250, 400, 600, and 250 W, respectively). After cooling, the digestion solutions were filtered with paper filters and diluted to 50 mL. The resulting solutions were analyzed by ICP-AES for the determination of Cd, Cr, Cu, Mn, Ni, Pb, and Zn.

## 3. Results and Discussion

### 3.1. Rice Husk Inorganic Characterization

Results obtained for the inorganic characterization of rice husk are reported in Table S3, expressed as mg/kg of rice husk. If all the inorganic components are considered, namely as ashes, it is up to 13.6% of the total mass. This is confirmed by Soltani et al. (2015), who found a value in the range of 15–20% and Bao (2023) in the range of 13–21% [35,36]. Concentrations are converted into their respective oxide and are compared with other studies in Table 1. The most abundant oxide is SiO_2_, constituting 88% of the rice husk ashes, followed by K_2_O (4.5%), CaO (2.1%), MgO (2.0%), Fe_2_O_3_ (1.5%), Al_2_O_3_ (1.3%), and Na_2_O (0.4%). The result for silica is consistent with other works that attest a value in the range of 67–97%. The presence of silica and consequently the existence of easily deprotonated silanol groups in rice husk is the main cause of the retention capacity towards heavy metals. As far as the other constituents are considered, the order of abundance is variable and could reflect the different rice families and crops specific to the regions considered. MnO_2_ and CuO were not determined in other studies, but in this work, they were found to be present in traces and count for 0.29% and 0.01% of the total ashes.

### 3.2. Heavy Metal Retention Tests

#### 3.2.1. Effect of Particle Size

**Figure 3:**
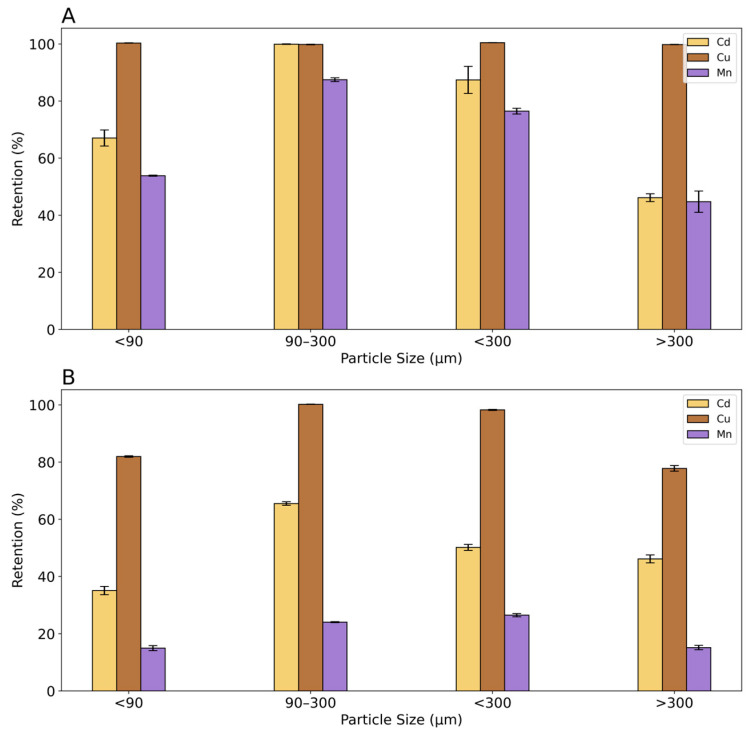
The percentage of retention of Cu, Cd, and Mn for the fractions of particle size: <90 µm; 90 µm–300 µm; <300 µm; >300 µm. (**A**) The retention with single metal experiments; (**B**) the retention with mixture metal experiments. Black bars represent confidence interval (95%).

Results of the effect of particle size on the retention of Cd, Cu, and Mn are shown in Figure 3. In the tests with individual metal solutions, Cu was retained with a value of 100% in all particle size fractions. Cd and Mn present a maximum retention in the fraction between 90 and 300, respectively, of 100% and 88%. A particle size fraction below 90 µm showed a 67% retention for Cd and 54% for Mn, whereas the fraction below 300 µm retained about 87% of Cd and 76% of Mn. The particle size fraction above 300 µm showed the lowest percent of retention, less than 50%, for both metals. Mn always shows the lowest % of retention in all particle size fractions. It can be concluded from this experiment that rice husk has a higher affinity for Cu, followed by Cd and Mn. Moreover, it is evident that the fraction with a grain size between 90 and 300 µm has the highest retention values.

The results of measurements with metal ion mixtures show that Cu has a highly competitive behavior and is retained to a greater extent than the other metals. Particle size also plays a significant role. Cu was quantitatively fixed with the particle size fraction between 90 and 300 µm whereas the adsorption is 98% and 80% with the <90 µm and >300 µm fraction, respectively. The adsorption of Cd and Mn in mixtures decreases compared to the values obtained with the individual metals, due to the intense competition of Cu. The highest Cd adsorption is in the fraction between 90 and 300 µm, and Mn adsorption in the fraction <300 µm and in the fraction between 90 and 300 µm is slightly lower.

From these results, it can be determined that the adsorption depends not only on particle size. The fraction >300 µm, having the lowest surface area, shows, as expected, the lowest absorption capacity. Nevertheless, low retention was observed also for the smallest particle size fraction (<90 µm), probably because of the difficulty in percolating the solution through the particle bed. Therefore, a dependence of retention on particle size can be observed for >90 µm for Cd and Mn, probably due to their lower affinity to cation exchange sites on the particle surface. Basing on these results, the particle size fraction between 90 and 300 µm was used for the subsequent tests.

Contaminant uptake will be increased with a larger surface area or smaller size of rice husk, as already reported in Shamsollahi et al. (2019) [38]. Many studies on rice husk as a biosorbent on heavy metals in the literature confirm its use in the particle size range found. A range between 250 and 297 µm was selected by Ajmal et al. (2003), 300 µm by Bishnoi et al. (2004), and 150 µm by Srivastava et al. (2006) [39,40,41].

#### 3.2.2. Effect of pH and Buffer Concentration

**Figure 4:**
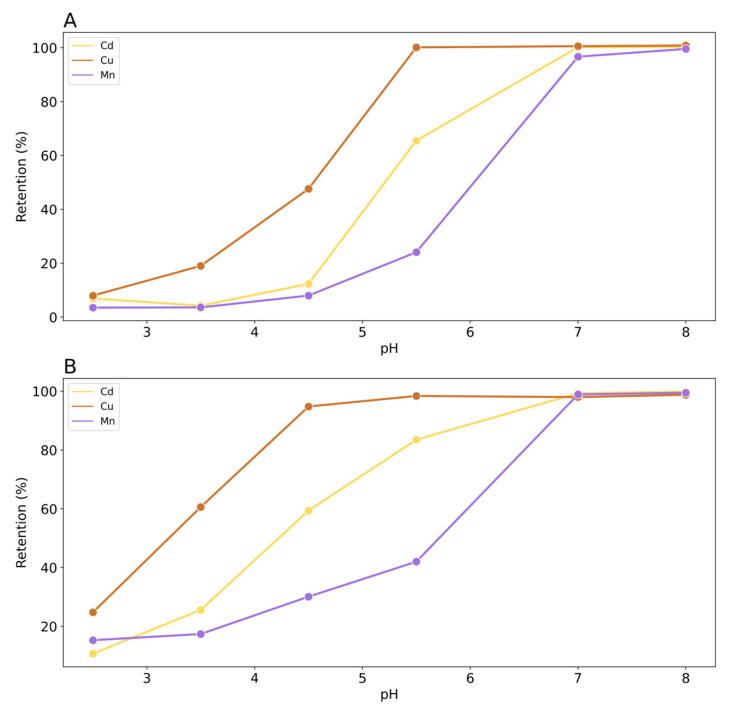
Retention of Cd, Cu, and Mn on rice husk as a function of pH (initial concentration of metals 1 × 10^−4^ M): (**A**) buffer concentration 1 × 10^−1^ M; (**B**) buffer concentration of 1 × 10^−2^ M.

Figure 4 shows the results of the effect of pH and buffer concentration on the retention of Cd, Cu, and Mn.

As expected, the retention of all metals decreased with the pH due to the progressive protonation of the cationic exchange sites present on the rice husk surface that are less available to retain the metals. Moreover, the retention decreases with the increasing of the buffer concentration, linked to the concentration of Na^+^, owing to the competition with other metals for adsorption sites. It is worth noting that in both cases of different ionic strengths, pH has a strong influence on metal retention. This means that the mechanism of interaction between rice husk and the metals could involve a formation of outer-sphere complexes that are pH-dependent [42]. It can be concluded that low buffer concentration is optimal for the retention of metals. Considering the different behavior of metals, Cu exhibits a retention of 99% at pH 5.5, regardless of the concentration of the buffer, followed by Cd, 64% and 32%, and Mn, 18% and 12%, for a buffer concentration of 1 × 10^−2^ M and 1 × 10^−1^ M, respectively. If we consider the hydroxide complex formation constant of the metals investigated in terms of pK_f_, we have Cu = 7.9; Cd = 10.1; and Mn = 10.6. This result may be indicative of a specific adsorption mechanism involving the exchange of metal cations with the surface ligands of the rice husk, which can partially form covalent bonds with these ions. In fact, the metals most able to form hydroxy complexes are specifically absorbed to the greatest extent [43]. But this is an explainable reason only for pH 5–8, where the hydroxy complexes are favored. However, the affinity series identified in the pH range of 5.5–8.0 does not appear to be related to the solubility of metal hydroxides. Indeed, according to the values of the solubility product constants (pK_sp_), Cu(OH)_2_ = 19.66; Cd(OH)_2_ = 13.60; and Mn(OH)_2_ = 12.72; pK_sp_ is only exceeded at pH 7.0 and pH 8.0 for Cu(OH)_2_ [44]. However, even at these pH levels, no precipitate was observed.

Considering only the interaction of metal aqueous species and silanol groups, the possibility of a mere electrostatic interaction can be ruled out, as the three metal cations are presented with the same charge. Meanwhile, if we consider the covalent index X_m_^2^r, where X_m_ is the electron-attracting capability of an atom in a molecule based on Pauling’s electronegativity and r is the ionic radius, the values for the three metals studied are in the following order: Cu^2+^ > Cd^2+^ > Mn^2+^. These results could again justify the order of retention exhibited in the experiments where Mn is the least retained followed by Cd and Cu.

#### 3.2.3. Effect of the Presence of Ligands

The behavior of heavy metals in the presence of organic ligands toward their adsorption on rice husk is crucial since organic compounds are typically present in natural waters, industrial and processing waters, soils, sediments, and wastewaters, in addition to heavy metals [45].

First, we chose a group of carboxylic acids with varying behaviors and increasing complexity: oxalic, malonic, succinic, glutaric, tartaric, and citric acids. We also took into account ligands that can be added to natural systems by anthropogenic activities, such as EDTA and NTA. For instance, the extensive use of EDTA as a chelate in industrial and agricultural settings raises the concentration of the chemical in a variety of water sources and wastewaters that could affect soils with accidental floods and spills [46].

Given the complexity of the system, the distribution of species in the solution under the experimental conditions used was calculated using PyEs software (Table S4). The main forms of the ligands at the pH studied are as follows: H_2_EDTA^2−^, HNTA^2−^, HTart^−^, HOxa^−^, HMal^−^, H_2_Succ, H_2_Glut and H_2_Citr^−^. For completeness, Table S5 summarizes the pKa of the ligands and the formation constant of the complexes between the metals and the ligands.

**Figure 5:**
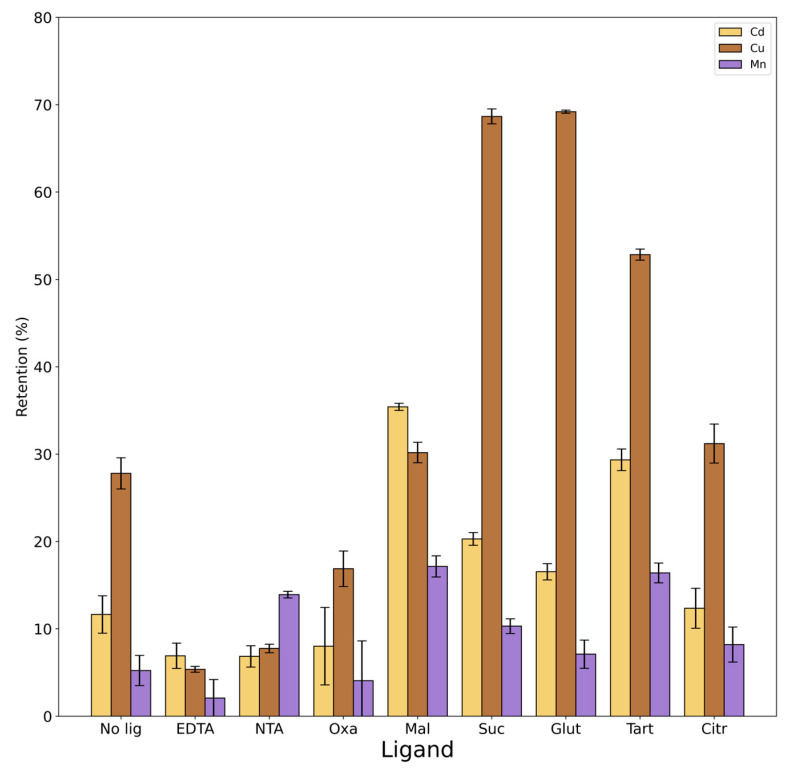
Percentages of retention of Cd, Cu, and Mn on rice husk obtained in the experiments with the different ligands compared to with the experiment with no ligand. Black bars represent confidence interval (95%).

Figure 5 shows the results obtained in the presence of the different ligands compared with the experiment led with the same conditions in the absence of ligands.

ANOVA results in Table S6 reported that for all metals, there was a statistically significant difference between groups. The presence of ligands with high complexation constants, such as NTA and EDTA, significantly prevents Cu from being retained by rice husk. On the other hand, the presence of NTA increases Mn retention by rice husk. Although the formation constant of the Mn-NTA complex has a higher value than all the complexes with other ligands (excluding EDTA), it is the lowest compared to Cd-NTA and Cu-NTA. If we consider the results of PyEs in Table S4, Mn in the presence of NTA is mainly free in the form of ions and there is no formation of the metal–ligand complex. This leads to the greater retention of Mn on the rice husk in the presence of that ligand.

Considering four dicarboxylic acids (oxalic acid, malonic acid, succinic acid, and glutaric acid), there is a slight correlation between the results and the length of their aliphatic chain. Ligands with smaller carbon chains (oxalic and malonic acids) have formation constants with all three metals higher than those of ligands with longer structures (succinic and glutaric acids). Therefore, the formation of metal complexes between smaller carboxylic acids and the metals should be favored and should lead to a decrease in the retention of the metal itself on the rice husk. In fact, if we report only statistically significant differences (*p* value < 0.01), Cu exhibits a retention value of 27% in the absence of ligands, while exhibiting values of 68 and 69% in the presence of succinic and glutaric acids, and a value of 17% in the presence of oxalic acid. This is confirmed by the highest percentage of free Cu in the presence of succinic and glutaric acid, also due to the fact that at a pH of 3.5, these ligands are not dissociated, whereas a formation of complexes like ML and ML_2_ with oxalic acid is observed that prevents the retention of Cu by rice husk.

Considering Mn and Cd with the four dicarboxylic acids, the formation of complexes between the metal and the ligands is not favored. In fact, both metals exhibit a slight increase in the retention on rice husk in the presence of ligands that is statistically significant (*p* value < 0.01) for Mn with malonic acid (from 5% to 17%) and for Cd with malonic acid (from 12% to 35%) and succinic acid (from 12% to 20%).

Although it appears that tartaric acid does not form complexes with any of the three metals investigated, experimental results show a significant increase in the retention with the presence of this ligand (from 5% to 17% for Mn, from 12% to 30% for Cd and from 27% to 52% for Cu). It cannot be ruled out that there may be an interaction between the rice husk exchange sites with the ligand in question that may facilitate greater metal retention in respect of the results given by the distribution of the species calculated by PyEs. In the study of Wong et al. (2003), in which different modifications of rice husk by various carboxilic acids (namely oxalic, malic, citric, and tartaric acids) were tested to improve metal sorptions from aqueous solutions, the results showed that the best were those obtained with rice husk modified with tartaric acid, shedding light on how this ligand interacts particularly with this matrix [47].

Finally, citric acid seems to not have an influence on the retention of the metals.

In summary, the results showed that the nature of the ligands affects the metal retention percentage on the rice husk. The effect of the ligands is different and depends on the structure of the ligands themselves but also on the type of metal, as well as on the pH of the system. Cu turns out to be the most variable among the three metals and the one that forms more stable complexes with all the ligands.

#### 3.2.4. Total Retention Capacity

After 150 mL of elution, the rice husk reached saturation with all the metals investigated. The breakthrough values show that the total retention capacity decreases in the following order: Cd (12.00 mg/g or 21.4 meq/100 g) > Cu (6.60 mg/g or 21.0 meq/100 g) > Mn (4.00 mg/g or 14.6 meq/100 g). This order is confirmed by a study of Krishnani et al. that investigated the capacity of alkali-treated rice husk on different metal ions adsorption [48]. They explained that lignin and cellulose, as major components of rice husk, have functional groups, i.e., alcohol, carboxyl, and ketone which interact with metallic cations. They found values of adsorption on rice husk of 14.4, 10.8, and 7.7 mg/g for Cd, Cu and Mn, respectively, for an initial concentration of the metal of 2.0 × 10^−4^ M at pH = 5.5. Other studies have focused on the conversion of rice husk into biochar for using it as an adsorbent for organic and inorganic pollutants. Bao (2023), Pellera et al. (2012), and Xu et al. (2013) found a value of 2.92 and 4.96 mg/g for Cu adsorption on biochar obtained from rice husk in the pH range 5–6 and an initial concentration of the metal of 0.1 M and 5.0 × 10^−3^ M [36,49,50].

Considering other types of materials that can be used as adsorbents, summarized in Table 2, it can be seen that eggshell wastes exhibit a higher capacity for the absorption of Cd and Cu than rice husk. On the other hand, the total retention capacity of rice husk towards Cd is greater than that of Na-montmorillonite and comparable to that of wheat straw and coal fly ash. The total retention capacity of rice husk for Cu is greater than that of Na–montmorillonite and wheat shell, but smaller than that of the other sorbents. In general, rice husk was found to have a good total capacity toward all considered metals.

### 3.3. Implementation in Semi-Field Conditions

#### 3.3.1. Effect on Mobility and Reactivity of Heavy Metals in Soil

To investigate the impact of rice husk on the mobility and reactivity of heavy metals in soil, we utilized a contaminated soil previously characterized by Malandrino et al. (2011) [28]. The contaminated soil exhibited notable contamination with heavy metals, surpassing regulatory thresholds established by “DECRETO 1 marzo 2019, n. 46” for agricultural soils in Italy [58]. Cr concentrations were found to be 3262 mg/kg, greatly exceeding the threshold limit of 150 mg/kg. Cu was 2777 mg/kg, surpassing the threshold of 200 mg/kg, while Ni levels reached 624 mg/kg, exceeding the limit of 120 mg/kg. Pb was also present at a concentration of 687 mg/kg, (threshold of 100 mg/kg), while the Zn content was 364 mg/kg, just above the threshold of 300 mg/kg.

In this previous work, the distribution of the heavy metals among the fractions obtained by applying Tessier’s protocol to Borgomanero soil was investigated. The results revealed higher concentrations of metals in the first two fractions in samples from the center of the site, indicating increased pollution levels. Particularly notable were the elevated percentages of Cd, Cu, Ni, Pb, and Zn in these fractions, attributable to anthropogenic sources. The large MgCl_2_-extractable fraction of these metals was influenced by the low soil pH. The percentages of metals in the first two fractions were significantly higher in the contaminated soil (Figure S2) compared to the control soil (Figure S3). Additionally, most heavy metals were extracted into the third fraction, with Cu being an exception, primarily found in the fourth fraction due to its complexation with organic matter. Cr behavior suggested a possible input in the environment as Cr(VI), a more mobile and toxic form, which could be reduced to Cr(III) in acidic soils, potentially adsorbed onto Fe oxides. Mn exhibited low extraction percentages in the first four fractions, indicating strong binding to the soil matrix and natural origin. This differentiation underscores the higher mobility of anthropogenic elements compared to lithogenic ones, suggesting potential environmental release under changing soil conditions [43]. The acidic environment found in this soil can significantly affect metal mobility. In fact, as pH decreases, the solubility of cationic species increases and H^+^ ions can compete with cations for binding sites in soil particles. Another critical factor is the exceptionally high organic matter content (30% *w*/*w*) of the contaminated soil. Repeated flooding from a nearby stream probably caused this accumulation. The original creek, now diverted, received industrial effluent, including that from electroplating plants. The high organic matter content can have a complex effect on metal mobility. On the one hand, organic matter can function as a ligand, immobilizing metals and reducing their availability. On the other hand, the decomposition of organic matter can also release complexing agents that can increase metal mobility improving their solubility also at higher pH values. Furthermore, the soil has a high cation exchange capacity (CEC) of 31.3 cmol/kg. CEC represents the ability of the soil to hold positively charged ions, including metals. A high CEC generally means a greater capacity to retain metals, potentially reducing their mobility and subsequent environmental risks.

**Figure 6:**
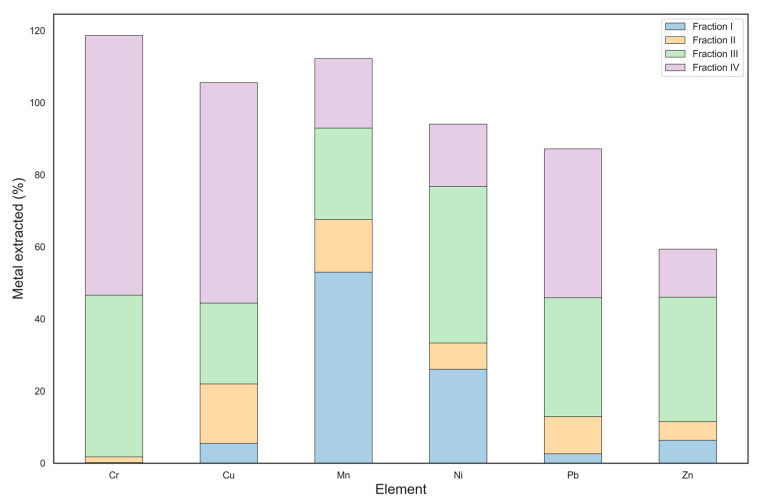
Heavy metal percentages extracted into the first four fractions according to Tessier’s protocol for contaminated soil amended by rice husk.

As shown in Figure 6, the addition of rice husk as a soil amendment resulted in a noticeable change in the distribution of heavy metals. A clear decrease in the concentration of Ni, Zn, and Pb was observed in the mobile fraction, suggesting that they may have been immobilized by rice husk. In addition to the high sorption capacity, rice husk can increase soil surface and porosity, further favoring metal retention and decreasing bioavailability to plant species. In addition, the introduction of rice husk displaced Cr and Cu into the strongly bound fractions compared to the unamended contaminated soil, likely due to chelation mechanisms with rice husk components. Finally, rice husk increased soil Mn mobility, probably due to the presence of Mn in the rice husk or to soil variability.

The Tessier sequential extraction was used to evaluate the availability of metals within the Borgomanero soil and to predict the uptake propensity of certain metals by plants. Significant positive correlations were found between the cumulative concentrations of Ni, Zn, and Pb in the initial two Tessier fractions and their corresponding concentrations in plant tissues (Ni − R = 0.83, *p* < 0.01; Zn − R = 0.97, *p* < 0.01; Pb − R = 0.87, *p* < 0.01). In contrast, Cu showed a weak positive correlation (R = 0.17, *p* > 0.05), suggesting that the fraction available to plants was not related to the first two Tessier fractions. Interestingly, there appeared a negative correlation for Mn (R = −0.43, *p* > 0.05) and no significant correlation was found for Cr (Cr − R = −0.09, *p* > 0.05), suggesting that the first two Tessier fractions may not accurately reflect plant-accessible Mn and Cr species. It is worth remembering that both metals can be present in soil at different oxidation states, influencing their mobility and bioavailability. It is possible that the first two Tessier fractions allow for a less accurate estimate of the pool available to plants for those metals that are mostly present in the soil in different ionic forms. These observations underscore the necessity for further research to refine the Tessier protocol or explore alternative methodologies to effectively measure the bioavailability of Cu, Mn, and Cr in soil.

#### 3.3.2. Effect on Lettuce and Spinach Heavy Metal Uptake

As shown in Table 3, lettuce and spinach plants grown in the heavy metal-contaminated soil had significantly higher metal concentrations than the control group grown in the uncontaminated soil. This indicates their ability to absorb and concentrate metals in their tissues under contaminated conditions. To evaluate the effectiveness of rice husk in decreasing the heavy metal uptake by vegetables, we calculated the “reduction efficiency” as follows: ([Me]C − [Me]RH)/[Me]C × 100. The incorporation of rice husk resulted in a significant reduction in heavy metal uptake by plants. In fact, by comparing the plants grown in the treated soils with the ones grown in the untreated contaminated soil, the reduction in heavy metal uptake ranged from 40 to 60% for Mn and Zn to almost complete elimination (close to 100%) for Cr, Cu, Ni, Cd, and Pb. These results are particularly noteworthy for Cr, Cu, and Ni, since their concentrations in the contaminated soil significantly exceeded the Italian limits.

Furthermore, the concentration of Pb, Cd, and Zn in the plants were found to be below the critical concentration limit for a 10% yield loss of the plant [43,59]. This is very important because it indicates that the concentration of these heavy metals does not result in a loss of crop yield. On the other hand, Ni, Cr, and Cu remained at levels of concern; however, the significant reduction observed compared to the initial highly contaminated conditions is a promising result. Specifically, the concentration of Cu in lettuce plants grown in soil amended with rice husk was similar to the baseline values found in the literature, demonstrating that rice husk may be an effective amendment for reducing Cu uptake by lettuce, confirming the high capability of the rice husk to retain Cu reported in the above sections [59].

The assessment of the plant capacity to absorb chemical elements from its growth medium, as determined by the Bioaccumulation Factor (BAF), showed that for Cd, BAF values exceeded 10, indicative of a pronounced propensity for accumulation within the plant, owing to the prevalence of mobile chemical forms that are readily accessible to plant uptake. In contrast, Zn, Cu, and Pb typically exhibit BAF values ranging between 1 and 0.1, signifying an intermediate level of accumulation within the plant tissue, attributable to the presence of chemical forms that are only moderately mobile. Meanwhile, Mn, Cr, and Ni typically display BAF values between 0.1 and 0.01, indicating a comparatively modest degree of accumulation within the plant, owing to the tendency of these metals to occur in forms that are relatively immobile and less accessible to plants (Cr and Ni) or present in large quantities in soils (Mn) [59].

**Figure 7:**
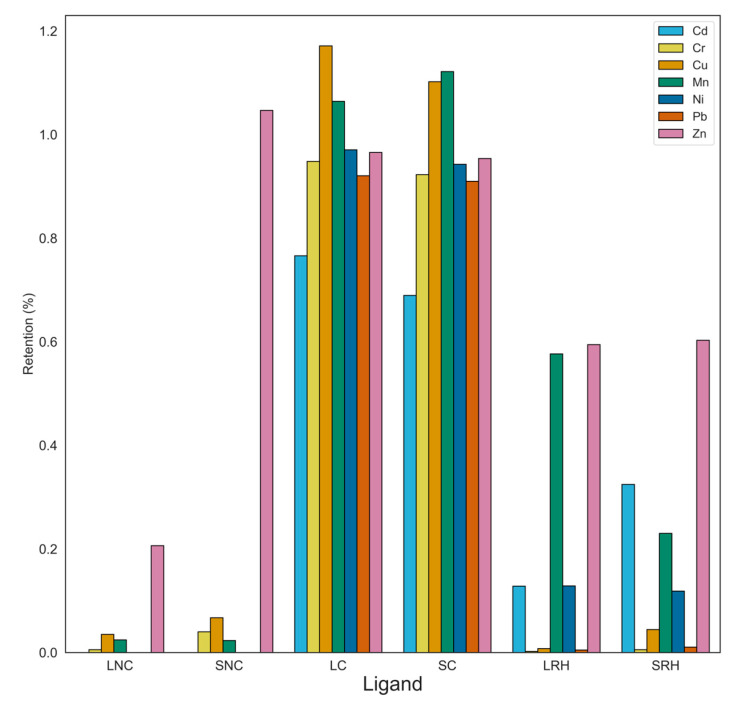
Bioaccumulation factors (BAFs) of heavy metals by plants, including data for lettuce and spinach cultivated under various conditions: in uncontaminated soil (LNC for lettuce, SNC for spinach), in contaminated soil (LC for lettuce, SC for spinach), and in contaminated soil amended with rice husk (LRH for lettuce, SRH for spinach).

As shown in Figure 7, BAF values were consistently below for plants grown in uncontaminated soils. This is likely due to the elevated organic matter content and substantial cation exchange capacity inherent to the control soil. Only Zn accumulated in spinach, which suggests an intrinsic predisposition of this plant toward the assimilation and accumulation of Zn. Plants cultivated in contaminated soil matrices typically exhibit BAF values approaching one, underscoring the accumulator behavior of lettuce and spinach in response to heightened concentrations and mobility of heavy metals within the soil environment.

The incorporation of rice husk as an amendment significantly reduces BAF values for most heavy metals. It is noteworthy that BAF values for Zn, Cr, and Ni return to baseline levels, due to the high immobilization determined by rice husk. Furthermore, BAF values for Cu, Pb, and Cd are significantly lower than the normal levels of bioaccumulation, indicating the effectiveness of chemical immobilization mechanisms in reducing the accumulation of these heavy metals in plant tissues [59]. Finally, the reduction in BAF values for Mn with the incorporation of rice husk is lower than those of other metals: this outcome may be explained by the lower affinity of Mn for rice husk, as explained in the previous sections of this study.

## 4. Conclusions

Rice husk has proven to be an efficient matrix in removing metal ions from aqueous solutions. Its retention depends on solution pH, buffer concentration, and the particle size of the rice husk. The highest retention efficiency was achieved with an intermediate rice husk particle size (90–300 µm), 1.0 × 10^−2^ M buffer concentration, and pH > 5.5. Organic acids that act as ligands may control the interaction of metals with the rice husk, depending on their nature and complex constants. In particular, this study quantitatively demonstrated that rice husk exhibits retention capacities of up to 100% for Cu, 64% for Cd, and 18% for Mn. This makes rice husk a promising and sustainable material for the amendment of contaminated soils, due also to its availability in massive quantities and at low cost.

The decrease in plant heavy metal uptake observed after rice husk application highlights its potential as a viable solution for mitigating metal pollution and safeguarding food safety. The pot experiments showed reductions in heavy metal uptake by *Lactuca sativa* and *Spinacia oleracea* of 40–60% for Mn and Zn, and nearly complete immobilization (up to 100%) of Cd, Cu, and Pb. Additionally, the modification of heavy metal distribution within soil fractions after amendment addition emphasizes the transformative role of rice husk in immobilizing metals and reducing their bioavailability. When compared with other common remediation media such as activated carbon or chemically treated biochar, rice husk offers a simpler, cost-effective alternative that is especially suitable for areas with significant agricultural production of rice, where this byproduct is abundant. However, it is important to note that while the rice husk was highly effective in immobilizing Cd, Cu, and Pb, its efficiency in reducing Mn mobility was lower, indicating a potential limitation for soils heavily contaminated with Mn. Additionally, although the pH-dependent nature of metal retention suggests broad applicability in soils with acidic to neutral pH, its performance in alkaline soils or soils with high organic matter content requires further investigation. However, further research is needed to refine remediation strategies and accurately predict metal bioavailability. Future studies should focus on long-term field trials to assess the durability of rice husk’s immobilization capacity under varying environmental conditions, such as rainfall, temperature fluctuations, and soil microbial activity. In conclusion, the chemical stabilization by rice husk evidenced in this study demonstrated to be a simple and cost-effective remediation technique that allows for the reduction in heavy metal assimilation from contaminated soils by edible plants and holds promise for sustainable agricultural practices applicable in urban and peri-urban soils.

## Figures and Tables

**Table 1:** Chemical composition in percentage (%) of rice husk ashes from various countries expressed as oxides.

	Vietnam ^1^	U.S.A. ^1^	Thailand ^1^	Nigeria ^1^	North Ireland ^1^	Malaysia ^1^	Japan ^1^	Iraq ^1^	India ^1^	Guyana ^1^	Canada ^1^	Brazil ^1^	Italy ^2^
SiO_2_	86.9	87–97	89–95	67–76	86–96	93.1	91.6	86.8	86–94	88–95	87–97	92.9	**87.8**
Al_2_O_3_	0.84	Traces	0.5–1.0	3–4.90	0.08–0.84	0.21	0.14	0.4	0.2–5.0	-	0.15–0.4	0.18	**1.3**
Fe_2_O_3_	0.73	0.38–0.54	2.5–2.8	0–0.95	0.03–0.73	0.21	0.06	0.19	0.3–2	-	0.16–0.4	0.43	**1.5**
CaO	1.4	0.25–1.0	1.0–1.3	1.36–6	0.3–1.4	0.41	0.58	1.4	0.5–2.5	0.06–1.2	0.4–0.49	1.03	**2.2**
K_2_O	2.46	0.58–2.0	2.4–2.5	0–0.1	0.7–2.4	2.31	2.54	3.84	0.1–2.3	0.6–2.5	2.0–3.0	0.72	**4.5**
M_g_O	0.57	0.12–2.0	0.18–0.28	1.3–1.81	0.1–0.5	1.59	0.26	0.37	0.1–1.8	0.17–0.26	0.35–0.50	0.35	**2.1**
Na_2_O	0.11	0–0.15	0.03–0.8	-	0.11–0.2	-	0.09	1.15	0.1–0.5	0–0.3	0.10–1.12	0.02	**0.4**
MnO_2_	-	-	-	-	-	-	-	-	-	-	-	-	**0.3**
CuO	-	-	-	-	-	-	-	-	-	-	-	-	**0.01**

^1^ [37], ^2^ This work.

**Table 2:** Total capacity calculated for Cd, and Cu expressed in mg/g for some materials.

	Total Capacity (mg/g)		References
	Cd	Cu	
Banana peel	30.7	49.5	[51]
Cashew nutshell	22.11	-	[52]
Wheat shell	-	0.83	[53]
Wheat straw	14.56	11.43	[54]
Eggshell waste	111.1	142.6	[55]
Coal fly ash	19.98	20.92	[56]
Na–montmorillonite	5.20	3.04	[57]
Rice husk	12	6.6	This work

**Table 3:** Concentrations of heavy metals (mg/kg dry weight) in plants, including data for lettuce and spinach cultivated in the control soil, in contaminated soil, and in contaminated soil amended with rice husk.

Plant	Metal	Control			Contaminated Soil			Contaminated Soil + Rice Husk			Reduction Efficiency
Lettuce	Cd	<0.004			2.85	±	0.30	0.38	±	0.02	87%
	Cr	0.18	±	0.047	3094	±	40	5.93	±	0.42	100%
	Cu	1.94	±	1.334	3254	±	215	18.6	±	15.2	99%
	Mn	6.14	±	0.393	289.2	±	7.5	171	±	17	41%
	Ni	0.97	±	0.43	606.5	±	25.5	68.7	±	0.64	89%
	Pb	<0.125			632.9	±	35.9	2.81	±	2.05	100%
	Zn	26.98	±	1.064	352.1	±	0.2	198	±	28.7	44%
Spinach	Cd	<0.004			2.57	±	0.27	0.97	±	0.03	74%
	Cr	1.327	±	0.022	3011	±	77	14.6	±	3.62	100%
	Cu	3.717	±	0.457	3062	±	6	109	±	3.39	98%
	Mn	5.844	±	0.154	304.9	±	8.3	68.3	±	7.23	59%
	Ni	1.288	±	0.291	589.1	±	6.7	63.4	±	0.43	89%
	Pb	<0.125			625.7	±	0.7	5.98	±	7.52	99%
	Zn	136.7	±	3.117	347.8	±	0.4	201	±	26.2	43%

## Data Availability

Data will be made available on request.

## Supplementary Materials

The following supporting information can be downloaded at: https://www.mdpi.com/article/10.3390/toxics12110790/s1, Table S1: Wavelengths chosen of the analytes determined and the limit of detections (*LOD*, µg/L); Table S2: Characteristics of the contaminated soil of Borgomanero and control soil; Table S3: Concentrations with standard deviation of the analytes determined in the rice husk, expressed in mg/kg; Table S4: Percentage of the formation of the species with the different ligands for Cu, Cd, and Mn, calculated by the software PyES. For each experiment the concentration of the metal is 1.0 × 10_−4_ M, the concentration of the ligand 3.0 × 10_−4_ M, and the concentration of the buffer acetate 1.0 × 10_−2_ M; Table S5: Equilibrium constants of the considered ligands [60,61,62]; Table S6: ANOVA table for each metal and its significance between the groups; Figure S1: A map of the location of the soils contaminated: on the top, the position of the site in the European continent; on the bottom, the position of the site in the Italian region; Figure S2: Heavy metal percentages extracted into the first four fractions according to Tessier’s protocol for contaminated soil; Figure S3: Heavy metal percentages extracted into the first four fractions according to Tessier’s protocol for non-contaminated soil.
