# Supplementary material for: Rice Husk as a Sustainable Amendment for Heavy Metal Immobilization in Contaminated Soils: A Pathway to Environmental Remediation
Source: Toxics. 2024 Oct 29;12(11):790. doi: 10.3390/toxics12110790 (PMC11597911; doi:10.3390/toxics12110790)
Supplement: Supplementary file 1 [file toxics-12-00790-s001.zip › toxics-3280876-supplementary.pdf]

toxics MDPI

Supplementary

Rice Husk as a Sustainable Amendment for Heavy Metal Immobilization in Contaminated Soils: A Pathway to Environmental Remediation

Riccardo Cecire [1,2], Aleandro Diana [1,*], Agnese Giacomino [3], Ornella Abollino [3], Paolo Inaudi [3], Laura Favilli [3], Stefano Bertinetti [1], Simone Cavalera [1], Luisella Celi [2] and Mery Malandrino [1,*]

[1] Department of Chemistry, University of Turin, Via Giuria 7, 10125 Turin, Italy; riccardo.cecire@unito.it (R.C.); stefano.bertinetti@unito.it (S.B.); simone.cavalera@unito.it (S.C.)
[2] Department of Agricultural, Forest and Food Sciences, University of Turin, Largo Paolo Braccini 2, 10095 Grugliasco, Italy; luisella.celi@unito.it
[3] Department of Drug Science and Technology, University of Turin, Via Giuria 9, 10125 Turin, Italy; agnese.giacomino@unito.it (A.G.); ornella.abollino@unito.it (O.A.); paolo.inaudi@unito.it (P.I.); laura.favilli@unito.it (L.F.)
* Correspondence: aleandro.diana@unito.it (A.D.); mery.malandrino@unito.it (M.M.)

Table S1. Wavelength chosen of the analytes determined and limit of detections *(LOD,* µg/L*).*

	Wavelength, nm	Limit of Detection, µg/L
Al	396.152	12
Ca	317.933	104
Cd	228.802	0.05
Cr	267.716	0.5
Cu	324.754	1
Fe	259.940	20
K	766.490	61
Mg	285.213	16
Mn	257.610	0.7
Na	589.592	83
Ni	232.604	0.9
Pb	220.802	0.08
Si	251.611	283
Sr	407.771	0.4
Zn	213.856	8

Table S2. Characteristics of the contaminated soil of Borgomanero and control soil.

Property	Contaminated Soil	Uncontaminated Soil
Organic carbon (% w/w)	17.14	34.00
Organic matter (% w/w)	29.55	59.02
CEC (cmol/kg)	31.30	57.22
pH	5.3	N.A.
Particle size distribution		
% Sand	19.3	49.03
% Fine sand	57.28	31.37
% Silt	10.1	6.28
% Fine silt	8.35	6
% Clay	5.37	8.12

Table S3. Concentrations with standard deviation of the analytes determined in the rice husk, expressed in mg/kg.

	**Concentration ± std. dev.**
Al	808 ± 24
Ca	1377 ± 41
Cd	< 2.5
Cu	3.9 ± 0.1
Fe	940 ± 27
K	2885 ± 82
Mg	1301 ± 42
Mn	185 ± 7
Na	272 ± 8
Si	55760 ± 1
Sr	12 ± 2

Table S4. Percentage of the formation of the species with the different ligand for Cu, Cd, Mn, calculated by the software PyES. For each experiment the concentration of the metal is $1.0\cdot10^{-4}$ M, the concentration of the ligand $3.0\cdot10^{-4}$ M and the concentration of the buffer acetate $1.0\cdot10^{-2}$ M.

	**Cu**	**Cd**	**Mn**
EDTA	(Cu)(EDTA)= 100	(Cd)(EDTA)= 99.8<br>Cd= 0.19<br>(Cd)(Ac)= 0.01	(Mn)(EDTA)= 63<br>Mn= 37<br>(Mn)(Ac)= 0.4
NTA	(Cu)(NTA)= 99.93<br>Cu= 0.07<br>(Cu)(Ac)= 0.01	Cd= 75<br>(Cd)(NTA)= 22<br>(Cd)(Ac)= 3	Mn= 98.5<br>(Mn)(Ac)= 1.2<br>(Mn)(NTA)= 0.3
Meso-tartaric acid	Cu= 90<br>(Cu)(Ac)= 7<br>(Cu)(Tart)= 3<br>(Cu)(Ac)$_2$= 0.05	Cd= 96<br>(Cd)(Ac)= 4<br>(Cd)(Tart)= 0.04	Mn= 98<br>(Mn)(Ac)= 1<br>(Mn)(Tart)= 0.7
Oxalic acid	(Cu)(Oxa)$_2$= 74<br>(Cu)(Oxa)= 15<br>Cu= 11<br>(Cu)(Ac)= 0.8<br>(Cu)(Ac)$_2$= 0.01	Cd= 94<br>(Cd)(Ac)= 4<br>(Cd)(Oxa)= 3	Mn= 98<br>(Mn)(Ac)= 1<br>(Mn)(Oxa)= 0.6
Malonic acid	Cu= 66<br>(Cu)(Mal)= 28<br>(Cu)(Ac)= 5<br>(Cu)(Ac)$_2$= 0.04	Cd= 96<br>(Cd)(Ac)= 4<br>(Cd)(Mal)= 0.2	Mn= 99<br>(Mn)(Ac)= 1<br>(Mn)(Mal)= 0.1
Succinic acid	Cu= 93<br>(Cu)(Ac)= 7<br>(Cu)(Suc)= 0.06<br>(Cu)(Ac)$_2$= 0.05	Cd= 96<br>(Cd)(Ac)= 4	Mn= 99<br>(Mn)(Ac)= 1
Glutaric acid	Cu= 93<br>(Cu)(Ac)= 7<br>(Cu)(Ac)$_2$= 0.05<br>(Cu)(Glu)= 0.04	Cd= 96<br>(Cd)(Ac)= 4<br>(Cd)(Glu)= 0.02	Mn= 99<br>(Mn)(Ac)= 1
Citric acid	Cu= 70<br>(Cu)(Citr)= 25<br>(Cu)(Ac)= 5<br>(Cu)(Ac)$_2$= 0.04	Cd= 94<br>(Cd)(Ac)= 4<br>(Cd)(Citr)= 0.2	Mn= 99<br>(Mn)(Ac)= 1<br>(Mn)(Citr)= 0.3

Table S5. Equilibrium constants of the considered ligands [60–62].

	Cd	Cu	Mn
EDTA<br>pK_{a1}= 2.00; pK_{a2}= 2.69;<br>pK_{a3}= 6.13; pK_{a4}= 10.19	A= 16.54<br>B=9.07	A= 18.7<br>B= 11.91<br>C= 6.70	A= 14.05<br>B= 5.47
NTA<br>pK_{a1}= 1.9; pK_{a2}= 2.48;<br>pK_{a3}= 9.65	A= 9.4<br>B= 4.9	A= 13.1<br>B= 3.39	A= 7.44<br>B= 3.55
Meso-tartaric acid<br>pK_{a1}= 2.97; pK_{a2}= 4.49	A= 1.30<br>B= 0.80<br>C= 0.76	A= 3.15<br>B= 2.06	A=2.49<br>B=1.41
Oxalic acid<br>pK_{a1}= 1.04; pK_{a2}= 3.8	A= 2.78<br>B= 1.22<br>C= 0.90<br>D= 4.1<br>E= 5.1	A= 4.49<br>B= 3.92<br>D= 9.54	A= 2.15<br>B= 1.90<br>C= 1.75
Malonic acid<br>pK_{a1}= 2.63; pK_{a2}= 5.28	A= 2.64<br>B= 1.49<br>C= 1.04	A= 5.04<br>B= 2.08<br>C= 7.8	A= 2.5<br>C= 1.24
Succinic acid<br>pK_{a1}= 4.0; pK_{a2}= 5.24	A= 1.47<br>B=0.82<br>C= 0.45<br>D= 2.29<br>E= 2.74	A= 2.7<br>B= 1.85	A= 1.26
Glutaric acid<br>pK_{a1}= 4.13; pK_{a2}= 5.01	A= 2.0	A=2.4	A= 1.13
Citric acid<br>pK_{a1}= 2.90; pK_{a2}= 4.35;<br>pK_{a3}= 5.30	A= 3.65<br>B=2.19<br>C=1.1<br>D=4.54	A= 5.90<br>B= 3.7<br>C=2.26	A= 3.79<br>B= 2.22<br>C=1.5

K_a=acid dissociation constant; β = cumulative stability constant. A = log β (ML); B = log β (MHL); C = log β (MH_2L); D = log β (ML_2); E = log β (ML_3).

Table S6. Anova table for each metal and their significance between the groups.

ANOVA Table		SS	df	F	PR(>F)	
Mn	Ligands	853	8	13,82	3,00E-06	***
	Residual	139	18			
Cd	Ligands	2504	8	77,99	2,18E-12	***
	Residual	72	18			
Cu	Ligands	13969	8	1031,62	2,40E-22	***
	Residual	30	18			

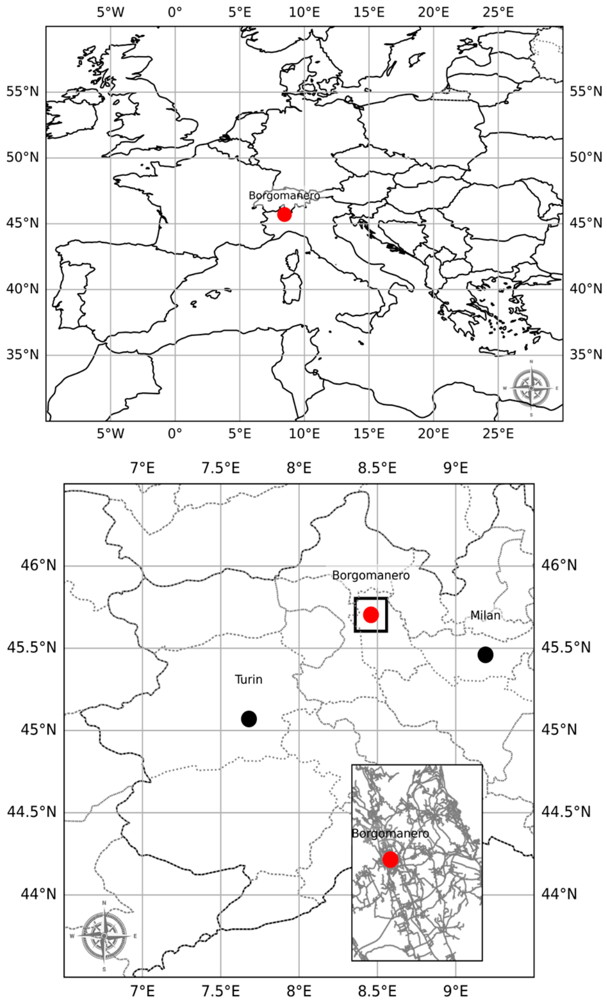

Figure S1. Map of the location of the soils contaminated, on the top the position of the site in the European continent; on the bottom the position of the site in the Italian region.

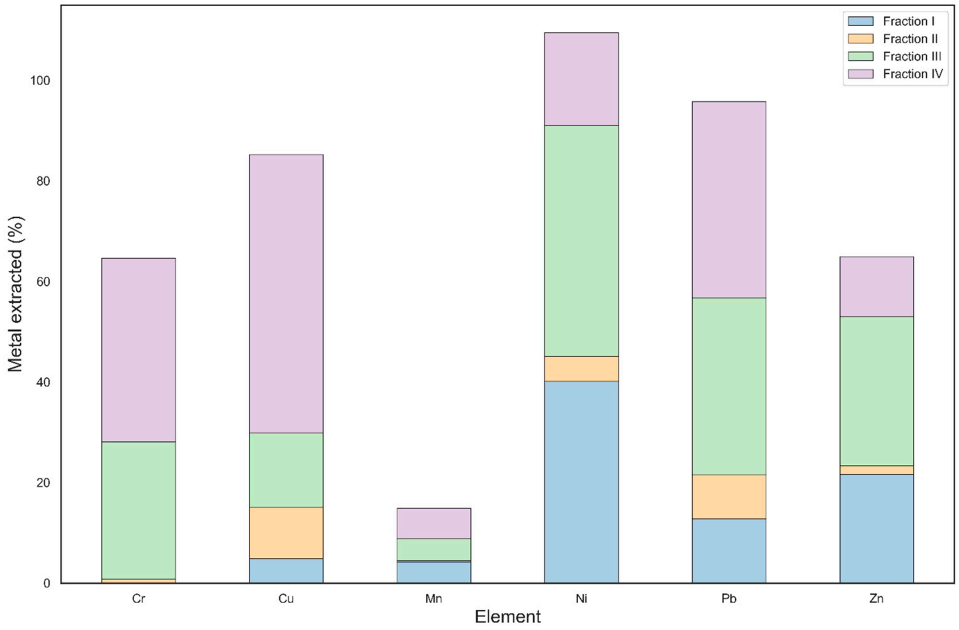

Figure S2. Heavy metal percentages extracted into the first four fractions according to Tessier's protocol for contaminated soil.

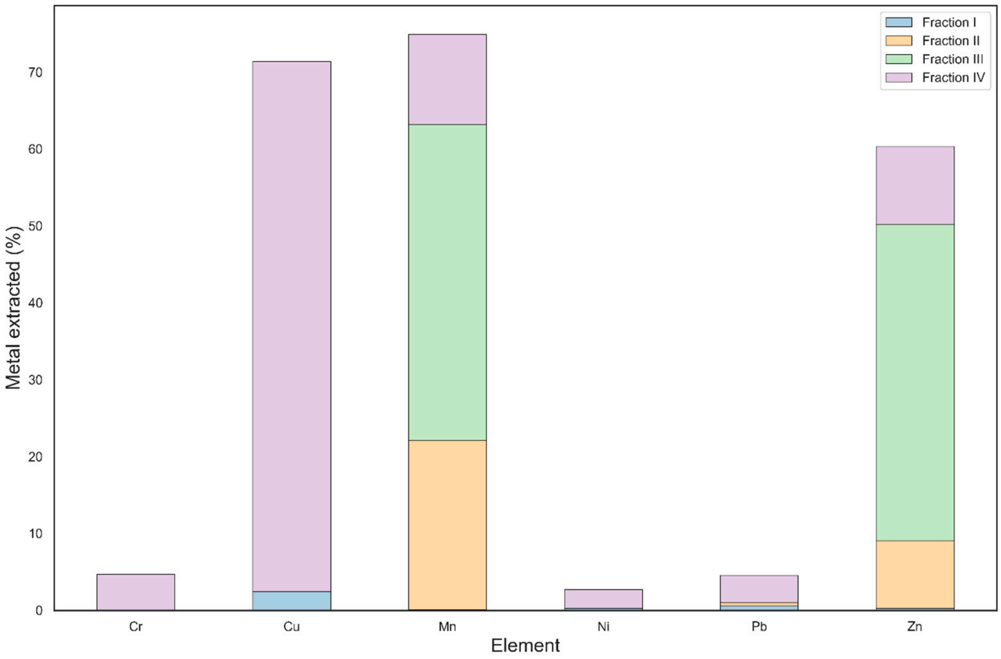

Figure S3. Heavy metal percentages extracted into the first four fractions according to Tessier's protocol for non-contaminated soil.
